# Towards tailored regimens in the treatment of drug-resistant tuberculosis: a retrospective study in two Italian reference Centres

**DOI:** 10.1186/s12879-019-4211-0

**Published:** 2019-06-28

**Authors:** Niccolò Riccardi, Riccardo Alagna, Laura Saderi, Maurizio Ferrarese, Paola Castellotti, Ester Mazzola, Saverio De Lorenzo, Pietro Viggiani, Zarir Udwadia, Giorgio Besozzi, Daniela Cirillo, Giovanni Sotgiu, Luigi Codecasa, for StopTB Italia Onlus Group

**Affiliations:** 1 Clinic of Infectious Diseases, IRCCS San Raffaele Scientific Institute, Milan, Italy; 2 StopTB Italia Onlus, Milan, Italy; 3 Emerging Bacterial Pathogens Unit, Division of Immunology, Transplantation and Infectious Diseases, IRCCS San Raffaele Scientific Institute, Milan, Italy; 4 Clinical Epidemiology and Medical Statistics Unit, Department of Medical, Surgical and Experimental Sciences, University of Sassari, Sassari, Italy; 5 E. Morelli Hospital ASST, Reference Centre for HIV-TB, Sondalo, Sondrio, Italy; 6 E. Morelli Hospital ASST, Reference Center for MDR-TB and HIV-TB, Sondalo, Italy; 7 Department of Pulmonary Medicine, P. D. Hinduja National Hospital and Medical Research Centre, Mumbai, Maharashtra India

**Keywords:** Pre-XDR-TB, Tuberculosis, DST, Individualized regimen

## Abstract

**Background:**

The increased incidence of drug-resistant TB is a major challenge for effective TB control. Limited therapeutic options and poor treatment outcomes of DR-TB may increase drug-resistance rates. The objective of the study is to retrospectively compare MDR-TB and pre-XDR-TB treatment regimens and outcomes in two large TB reference centres in Italy from January 2000 to January 2015.

**Methods:**

A retrospective, multicentre study was conducted at the Regional TB Reference Centre Villa Marelli Institute (Milan) and at the Reference Center for MDR-TB and HIV-TB, Eugenio Morelli Hospital (Sondalo). The supra-national Reference Laboratory in Milan performed DST. Inclusion criteria were: age ≥ 18 and culture-confirmed diagnosis of MDR- or pre-XDR TB. Chi-square or Fisher exact test was used to detect differences in the comparison between treatment outcomes, therapeutic regimens, and drug-resistances. Computations were performed with STATA 15.

**Results:**

A total of 134 patients were selected. Median (IQR) age at admission was 33 (26–41) years and 90 patients (67.2%) were male. Pulmonary TB was diagnosed in 124 (92.5%) patients. MDR- and pre-XDR-TB cases were 91 (67.9%) and 43 (32.1%), respectively. The WHO shorter MDR-TB regimen could have been prescribed in 16/84 (19.1%) patients. Treatment success was not statistically different between MDR- and pre-XDR-TB (81.3% VS. 81.4%; *P* = 0.99). Mortality in MDR-TB and pre-XDR-TB groups was 4.4 and 9.3%, respectively (*P* = 0.2). Median duration of treatment was 18 months and a total of 110 different regimens were administered. Exposure to linezolid, meropenem, and amikacin was associated with a better outcome in both groups (*P* = 0.001, *P* < 0.001, and *P* = 0.004, respectively).

**Conclusions:**

Tailored treatment regimens based on DST results can achieve successful outcomes in patients with pre-XDR-TB.

## Background

Approximately 10 million people infected by *Mycobacterium tuberculosis* (MTB) develop tuberculosis (TB) disease annually. TB is recognized as the leading cause of death from an infectious agents [1]. Even if globally TB mortality rate is decreasing at an annual rate of about 3%, the increased incidence of multi-drug-resistant TB (MDR-TB) represents a major challenge for effective TB control, undermining the goals of the End TB strategy for 2035 [2]. The World Health Organization (WHO) defines pre-extensively drug-resistant TB (pre-XDR-TB) a TB form caused by MTB strains with resistance to rifampicin (RMP), isoniazid (INH) (MDR-TB) and a second-line injectable agent (SLIs) or to any fluoroquinolone (FQ), whereas extensively drug-resistant TB (XDR-TB) is caused by a MTB strain resistant to INH, RMP, at least one SLIs agent and to any FQ [3]. Although, 600,000 RMP-resistant (RR) and MDR-TB cases were estimated globally in 2016, epidemiology of pre- and XDR-TB is scarce [1]. In fact, only 28% of the estimated DR-TB cases are notified [3, 4]. Currently, the majority of the DR-TB cases occur in Eastern Europe and central Asia [3, 4]. Migration from high to low TB incidence countries has recently contributed to increase the burden of resistant TB cases in countries of arrival [5–7]. Indeed, a total of 2.8% (range: 1.8–4.3%) and 13% (range: 7.7–21%) of all new and previously treated TB cases showed drug resistance patterns in Italy, a low TB incidence country [1].

Limited therapeutic options, adherence and complexity of the regimens associated with the currently available treatments for MDR/XDR-TB may increase drug-resistance rates [4–8]. Although an updated drugs hierarchy for treating patients with MDR-TB has been recently released, knowledge on the efficacy of WHO-recommended regimens for complicated MDR-TB is poor and there is little-to-none evidence on the best therapeutic regimens for pre- and XDR-TB [9–12].

The objective of the present study is to retrospectively compare MDR and pre-XDR-TB treatment regimens, as well as treatment outcomes, of two large TB reference centres located in Northern Italy during a 15-year period.

## Methods

A retrospective study was carried out in two Italian TB reference centres (TB Reference Centre of Lombardy Region, Villa Marelli Institute/ASST Niguarda Ca′ Granda, Milan, and at the Reference Center for MDR-TB and HIV-TB, Eugenio Morelli Hospital ASST, Sondalo, Italy). Villa Marelli Institute is an outpatient reference center for drug-susceptible and RR/MDR/pre-XDR/XDR-TB, serving a population of more than 10 million people and dealing with ~ 350 patients per year, of whom ~ 3% with DR-TB [13]. At the Villa Marelli Institute outpatients are diagnosed, treated and followed-up with ambulatory care. The Eugenio Morelli Hospital is the national inpatient reference center for DR-TB and HIV-TB co-infection and deals with ~ 225 TB patients annually, of whom ~ 7.5% with DR-TB. At the Eugenio Morelli Hospital, all admitted cases are hospitalized until culture conversion and clinical stability are achieved [14]. Only at E.Morelli Hospital patients are hospitalized in case of severe manifestation of the disease (e.g. meningitis, pericarditis), while the Villa Marelli Institute works as an outpatient service for TB patients that do not require hospitalization, regardless of the resistance pattern (e.g. clinically stable patient with pulmonary pre-XDR-TB that can be effectively in isolation at home). Patients can be referred at both centres from other Hospitals, General Practitioners, screening program for at risk populations or walk-in consultation. As reference centres, at both Institutions the drugs available are the same. The Regional Reference Laboratory in Milan carried out the drug-susceptibility test (DST), whose quality is ensured by a once-a-year supranational proficiency testing, performed according to international standards [15]. Patient selection criteria were: age ≥ 18 years, MDR- or pre-XDR TB, availability of required microbiological, radiological, and laboratory data. Data of patients notified from 1st of January 2000 to 1st of January 2015 were collected. The following information were retrieved: demographic (age at admission, sex, nationality), epidemiological and clinical (risk factors for TB disease, HIV status, localization of the disease), radiological (at the admission and at the end of treatment), bacteriological (smear, culture, NAAT, DST; smear and culture results at 30 days, 60 days, 90 days from the beginning of treatment and at the end of treatment) and treatment variables. At both sites, a standardized method based on clinical evaluation was used to record adverse events.

**Fig. 1:**
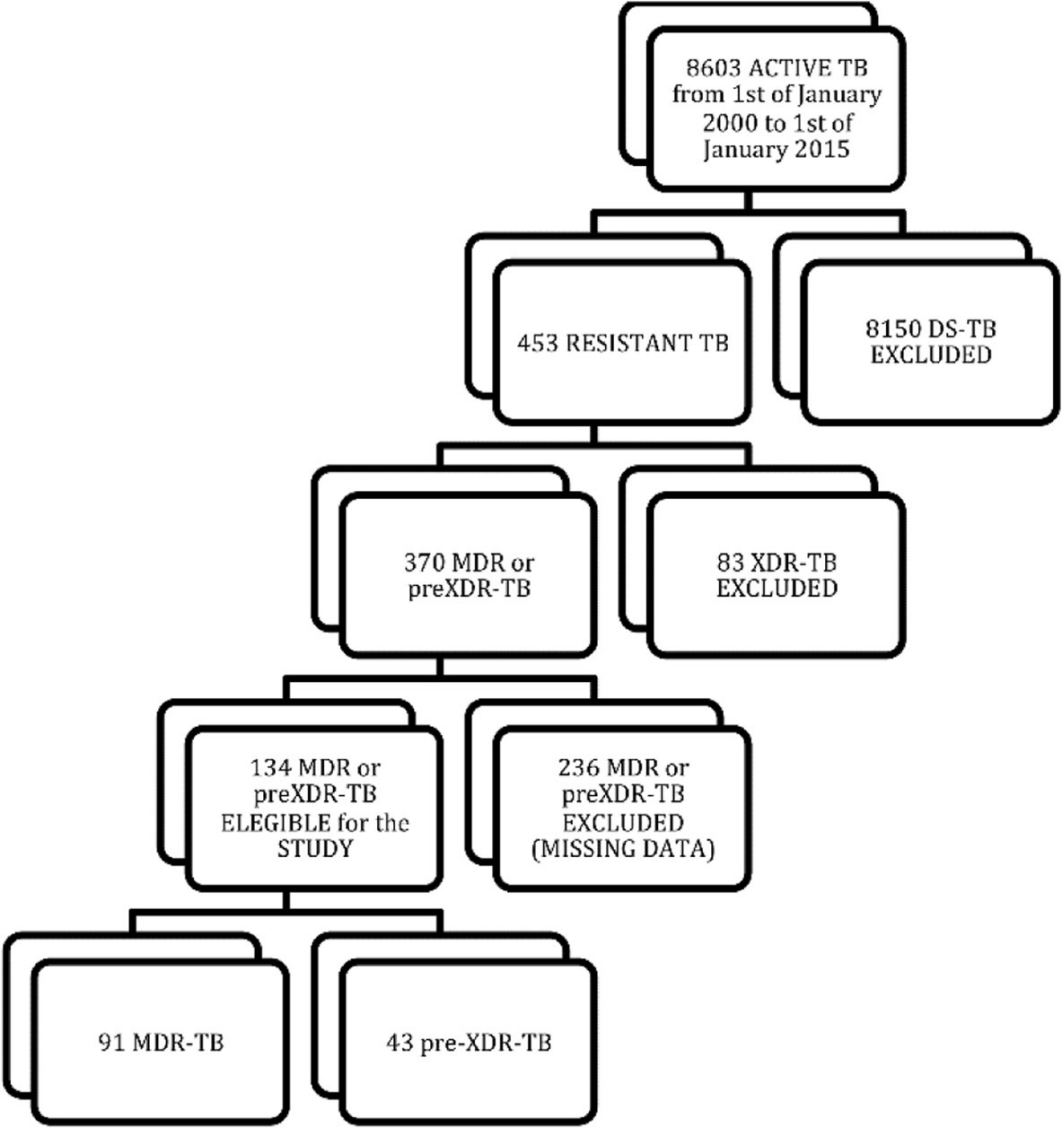
Patients’ selection flow-chart

The flowchart in Fig. 1 shows patients’ selection cascade.

Sputum smear examinations were performed weekly until negative and then monthly. Cultures were performed monthly both while sputum smear positive and negative. Patients were started on HRZE standard regimen until the result of the DST was available, if no previous contact with MDR-TB were known or no rpoB mutation was detected by Xpert MTB/RIF. If patients were contact of known MDR-TB treatment and the contact’s DST was available, they were started on the same treatment of the contact until DST results. If there was no known contact and Xpert MTB/RIF reported the presence of R-resistance, treatment for MDR-TB according to WHO guidelines [16] was started and, when the DST results were available, the treatment was individualized according to it. At both Centres, DST based and patients’ centred-treatments were designed.

Treatment outcomes based on 2016 WHO criteria were recorded [15]. Sputum conversion was defined as two consecutive negative sputum smears in patients who were sputum smear-positive at diagnosis. Time to culture conversion was defined as time from treatment start to the date of the first of two consecutive negative cultures [17]. The primary outcome measure was the proportion of patients with favorable treatment outcome (cured and treatment completed). Secondary outcomes were comparison of treatment outcomes between specific drug-containing regimens and possible eligibility for WHO shorter MDR-TB regimen [18]. Based on its observational and retrospective epidemiological nature, only some patients underwent a complete bacteriological assessment; then, the denominators changed overtime for all outcomes. Adverse events leading to discontinuation of the drugs were recorded.

This study was reviewed and approved by the ethical committee of the coordinating centre of ASST Niguarda Ca′ Granda in Milan (Italy) (Registration number: 578–112,018). As a retrospective observational study, the ethical committee waived the need to obtain written informed consent and allowed us to use the information (previously collected) from our database. STROBE recommendations were followed.

### Statistical analysis

An ad hoc electronic form was used to collect demographic, epidemiological, clinical, and microbiological variables. Qualitative variables were summarized with absolute and relative (percentages) frequencies, whereas quantitative variables were summarized with means (standard deviations, SD) or medians (interquartile ranges) based on their parametric distribution, respectively. Chi-square or Fisher exact tests were used to assess statistical differences for qualitative variables; student t-test or Mann-Whitney test were used for parametric and non-parametric variables. A two-tailed *p*-value less than 0.05 was considered statistically significant. All statistical computations were performed with the statistical software STATA version 15 (StataCorp, Texas, US).

## Results

### Demographic parameters

A total of 134 patients were included in the analysis, 89 (66.4%) from Villa Marelli Institute and 45 (33.6%) from Eugenio Morelli Hospital. Between 2000 and 2008, 56 (41.8%) patients were enrolled in the study, whereas 78 (58.2%) between 2009 and 2015.

Median (IQR) age at admission was 33 (26–41) years, 90 (67.2%) patients were male.

Resistance type was MDR-TB in 91(67.9%) patients and pre-XDR-TB in 43(32.1%) patients.

Foreign-born patients were 116 (86.6%) and the most represented WHO area was the European Region with 81 (60.5%) patients, followed by the American Region with 23 (17.2%) and by the African Region with 13 (9.7%) cases. The most represented nationality was Romanian with 30 (22.4%) patients, followed by Italian with 18 (13.4%), by Ukrainian and Peruvian with both 17 (12.7%) patients.

Foreign-born patients represented the majority of the pre-XDR-TB cases (34/43, 79%) and they came from the WHO European Region in 22 cases (64%), while the Eastern Mediterranean region had the highest prevalence with 5 out of 10 patients (50%) followed by South-East Asia Region (3 patients, 37.5%). The most represented nationalities with the pre-XDR-TB were Romanian, Italian, Ukrainian and Indian with 13 (30.2%), 9 (20.9%), 7 (16.2%) and 3 (7%) patients, respectively.

The main known risk factors for TB were: a previous contact with a TB patient in 17 (34.0%) cases, HIV infection in 13 (26%), and diabetes in 5 (10.0%) patients. Five out 13 (38.3%) patients were not on cART, while 4/13 (30.7%) on tenofovir disoproxil fumarate/emtricitabine/lopinavir/ritonavir, 3/13 (23%) tenofovir disoproxil fumarate/emtricitabine /atazanavir/ritonavir and 1/13 (8%) on tenofovir disoproxil fumarate/emtricitabine /dolutegravir once daily (no use of rifampicin). Unfortunately, CD4+ cell count and VL were not available.

### Clinical and radiological parameters

A According to the WHO definitions, 63 (47.0%) of patients included in the study had a new diagnosis in 63 (47.0%) cases, whereas in 48 (35.8%) cases a previous treatment failure was documented, 17 (12.7%) cases were relapse, and chronic TB was found in 6 (4.5%) cases. Pulmonary TB (PTB) was diagnosed in 124 (92.5%) patients and bilateral pulmonary involvement with cavitary lesions was found in 40 (40.4%) patients, followed by cavitary lesions affecting only one lung and bilateral pulmonary involvement without cavitary lesions in 38 (38.4%) and 11 (11.1%) patients, respectively. A non-cavitary and non-bilateral radiological pattern was showed in 10 (10.0%) patients. Among the 19 (14.2%) extra-pulmonary TB (EPTB) cases, the most frequently involved organs were peripheral lymph nodes and pleurae in 9 (60.0%) and 3 (20%) patients, respectively. Ten patients had PTB alone and 9 both EPTB and PTB. EPTB was diagnosed by culture on biopsy and the treatment outcomes were assessed on clinical response.

### Mycobacteriological and resistance parameters

Sputum smear and culture positivity was recorded in 102 (76.1%) and 128 (95.5%) patients, respectively. Six MDR-TB contact-cases were treated without microbiological confirmation based on the high clinical and radiological suspicion. Resistance patterns are showed in Table 1. Median (IQR) time to sputum smear conversion was 42 (21–61) days, while median (IQR) time to culture conversion was 37.5 (19.0–59.0) days. Smear and culture negativity at the end of treatment were achieved in 86/88 (97.7%) and 83/88 (94.3%) cases, respectively.

**Table 1:** Drug resistance patterns in the included sample

*Resistance to rifampicin, n (%)*	134 (100.0)
*Resistance to isoniazid, n (%)*	134 (100.0)
*Resistance to ethambutol, n (%)*	65 (48.5)
*Resistance to pyrazinamide, n (%)*	64 (48.5)
*Resistance to streptomycin, n (%)*	86 (64.2)
*Resistance to amikacin, n (%)*	14 (11.4)
*Resistance to fluoroquinolones, n (%)*	29 (21.6)
*Resistance to ethionamide, n (%)*	44 (50.6)
*Resistance to cycloserine, n (%)*	24 (33.3)
*Resistance to PAS, n (%)*	26 (29.2)
*Resistance to linezolid, n (%)*	2 (2.7)

### Prescribed regimens

The most commonly used drugs in the study population were as follow: FQ exposure was recorded in 119 (88.8%) cases, amikacin exposure in 65 (48.5%), linezolid exposure in 46 (34.3%), meropenem exposure in 45 (33.6%), and clofazimine exposure in 25 (18.7%). Median (IQR) duration of treatment was of 18 [18–20] months.

Adverse events were reported in 26 (19.6%) patients; 7/46 (15.2%) and 14/65 (21.5%) discontinued linezolid and SLIs owing to severe adverse events, respectively. Even if not applicable for pre-XDR-TB, with a mean number of 2 (SD 1.4) resistances to the drugs included in the WHO shorter MDR-TB regimen, prescription of the shorter regimen would have been implemented in 16/84 (19%) patients with available DST for all the drugs composing the regimen (Table 2).

**Table 2:** Resistance to the drugs composing the World Health Organization shorter MDR-TB regimen

*Resistance to amikacin, n (%)*	14/123 (11.4)
*Resistance to fluoroquinolones, n (%)*	29/134 (21.6)
*Resistance to ethionamide, n (%)*	44/87 (50.6)
*Resistance to pyrazinamide, n (%)*	64/132 (48.5)
*Resistance to ethambutol, n (%)*	65/134 (48.5)
*Susceptibility to all drugs included, n (%)*	16/84 (19.1)
*Resistance to all drugs included, n (%)*	0/84 (0.0)
*Mean (SD) no. of resistances*	2 (1.4)
*Resistance to pyrazinamide, ethambutol, ethionamide, fluoroquinolones, n (%)*	10/86 (11.6)
*Resistance to pyrazinamide, ethambutol, ethionamide, kanamycin, n (%)*	5/84 (6.0)
*Resistance to pyrazinamide, ethambutol, ethionamide, n (%)*	25/86 (29.1)
*Resistance to pyrazinamide, ethambutol, fluoroquinolones, n (%)*	14/132 (10.6)
*Resistance to pyrazinamide, ethambutol, kanamycin, n (%)*	8/121 (6.6)
*Resistance to pyrazinamide, ethambutol, n (%)*	47/132 (35.6)

### Treatment outcome

Overall treatment success was achieved in 109 (81.3%) cases. Treatment success did not statistically differ between MDR-TB 74 (81.3%) and pre-XDR-TB 35 (81.4%) (*P* = 0.99). Mortality in MDR- and pre-XDR-TB groups was 4 (4.4%) and 4 (9.3%), respectively (*P* = 0.27).

Exposure to linezolid, meropenem, and amikacin in the treatment regimens was associated with a better outcome (*P* = 0.001 for linezolid, *P* < 0.001 for meropenem, and *P* = 0.004 for SLIs), whereas exposure to FQ and clofazimine was not statistically significant (*P* = 0.33 and *P* = 0.13, respectively). In the sub analysis of FQ-resistant patients that were exposed to FQ vs not exposed group, treatment success was not statistically different (*P* = 0.35). The most administered anti-TB regimen was composed by moxifloxacin, ethambutol, terizidon and ethionamide (in 10 patients, 7.4%), and 5 (3.7%) patients had the same regimen in addition to pyrazinamide. Meropenem/clavulanic acid, cicloseryne, clofazimine, linezolid and para-aminosalicylic acid were prescribed as anti-TB regimen in 5 (3.7%) cases. The regimen based on terizidone, para-aminosalicylic acid, moxifloxacin, linezolid and bedaquiline was administered in two (1.5%) patients as well as linezolid, amikacin, ethionamide and moxifloxacine (1.5%). However, 110 different regimens were administered (at least one different drug, not of the same class, in the regimen) according to the result of DSTs. Tables 3 and 4 show the comparison of treatment outcomes between specific drug-containing regimens. Thirty-day treatment culture negativity was reached in 42 (50.0%) patients, while 60-and 90-day treatment culture negativity in 65 (77.4%) and 73 (86.9%) patients, respectively. Culture negativity at the end of treatment occurred in 83 (94.3%) patients that were culture positive at the beginning of treatment. Median (IQR) time to culture conversion was of 37.5 (19.0–59.0) days. Improvement of radiological signs was detected in 67 (84.8%) patients.

**Table 3:** Comparison of treatment outcomes between specific drug-containing regimens

		Not exposed	Exposed	p-value
Clofazimine-containing regimens		(*n* = 109)	(*n* = 25)	
*Treatment outcome, n (%)*	*Cured*	45 (41.3)	12 (48.0)	0.13
	*Treatment completed*	44 (40.4)	8 (32.0)	
	*Died*	4 (3.7)	4 (16.0)	
	*Default*	9 (8.3)	1 (4.0)	
	*Transferred out*	7 (6.4)	0 (0.0)	
Linezolid-containing regimens		(*n* = 88)	(*n* = 46)	
*Treatment outcome, n (%)*	*Cured*	28 (31.8)	29 (63.0)	0.001
	*Treatment completed*	41 (46.6)	11 (23.9)	0.01
	*Died*	4 (4.6)	4 (8.7)	0.44
	*Default*	9 (10.2)	1 (2.2)	0.16
	*Transferred out*	6 (6.8)	1 (2.2)	0.42
Meropenem-containing regimens		(*n* = 89)	(*n* = 45)	
*Treatment outcome, n (%)*	*Cured*	25 (28.1)	32 (71.1)	< 0.0001
	*Treatment completed*	44 (49.4)	8 (17.8)	< 0.0001
	*Died*	3 (3.4)	5 (11.1)	0.12
	*Default*	10 (11.2)	0 (0.0)	0.02
	*Transferred out*	7 (7.9)	0 (0.0)	0.10
Fluoroquinolones-containing regimens		(*n* = 15)	(*n* = 119)	
*Treatment outcome, n (%)*	*Cured*	5 (33.3)	52 (43.7)	0.33
	*Treatment completed*	8 (53.3)	44 (37.0)	
	*Died*	2 (13.3)	6 (5.0)	
	*Default*	0 (0.0)	10 (8.4)	
	*Transferred out*	0 (0.0)	7 (5.9)	
Amikacin-containing regimens		(*n* = 69)	(*n* = 65)	
*Treatment outcome, n (%)*	*Cured*	21 (30.4)	36 (55.4)	0.004
	*Treatment completed*	31 (44.9)	21 (32.3)	0.13
	*Died*	4 (5.8)	4 (6.2)	1.0
	*Default*	9 (13.0)	1 (1.5)	0.02
	*Transferred out*	4 (5.8)	3 (4.6)	1.0

**Table 4:** Comparison of treatment outcomes between specific drug-containing regimens by drug-resistance pattern (MDR- VS- pre-XDR TB)

		MDR-TB	Pre-XDR TB	p-value
Clofazimine-containing regimens		n = 8	*n* = 17	
*Treatment outcome, n (%)*	*Cured*	5 (62.5)	7 (41.2)	0.41
	*Treatment completed*	2 (25.0)	6 (35.3)	1.0
	*Died*	1 (12.5)	3 (17.7)	1.0
	*Default*	–	1 (5.9)	1.0
	*Transferred out*	–	–	–
	*Treatment success*	7 (87.5)	13 (76.5)	1.0
Linezolid-containing regimens		*n* = 23	*n* = 23	
*Treatment outcome, n (%)*	*Cured*	17 (73.9)	12 (52.2)	0.22
	*Treatment completed*	3 (13.0)	8 (34.8)	0.17
	*Died*	1 (4.4)	3 (13.0)	0.61
	*Default*	1 (4.4)	–	1.0
	*Transferred out*	1 (4.4)	–	1.0
	*Treatment success*	20 (87.0)	20 (87.0)	1.0
Meropenem-containing regimens		*n* = 22	*n* = 23	
*Treatment outcome, n (%)*	*Cured*	20 (90.9)	12 (52.2)	0.007
	*Treatment completed*	0 (0.0)	8 (34.8)	0.004
	*Died*	2 (9.1)	3 (13.0)	1.0
	*Default*	–	–	
	*Transferred out*	–	–	
	*Treatment success*	20 (90.9)	20 (87.0)	1.0
Fluoroquinolones-containing regimens		*n* = 88	*n* = 31	
*Treatment outcome, n (%)*	*Cured*	39 (44.3)	13 (41.9)	0.82
	*Treatment completed*	33 (37.5)	11 (35.5)	0.84
	*Died*	3 (3.4)	3 (9.7)	0.18
	*Default*	9 (10.2)	1 (3.2)	0.45
	*Transferred out*	4 (4.6)	3 (9.7)	0.38
	*Treatment success*	72 (81.8)	24 (77.4)	0.59
Amikacin-containing regimens		*n* = 43	*n* = 22	
*Treatment outcome, n (%)*	*Cured*	28 (65.1)	8 (36.4)	0.03
	*Treatment completed*	11 (25.6)	10 (45.5)	0.11
	*Died*	2 (4.7)	2 (9.1)	0.60
	*Default*	1 (2.3)	–	1.0
	*Transferred out*	1 (2.3)	2 (9.1)	0.26
	*Treatment success*	39 (90.7)	18 (81.8)	0.43

## Discussion

This study represents, to the best of our knowledge, the largest sub-group of pre-XDR-TB in Italy. The nationalities of patients with MDR-TB born outside Italy reflects previous migration trends to Italy in the past 30 years, and estimated prevalence of MDR-TB in the patients’ countries of origin [1, 3, 8]. However, the high number of pre-XDR-TB born in Italy may be related to easier access to screening and to reference centres for native population [4].

The high successful outcome rate for both MDR and pre-XDR-TB could be explained by the following factors: drugs availability, reliability of microbiological results and expertise in managing difficult-to-treat TB cases [19]. In fact, all collected samples were tested for resistances to anti-TB agents according to the current WHO TB treatments guidelines in those years. Nevertheless, minimal inhibitory concentration testing and molecular susceptibility tests, not available at the time of the study, can currently offer another key diagnostic tool to improve treatment management. Accurate DST methods helped diagnose pre-XDR-TB patients, allowing the prescription of a high number of tailored regimens. No differences in terms of clinical outcomes were found between MDR- and pre-XDR TB patients; however, higher mortality rate, even if not statistically significant, was recorded in pre-XDR TB cases, highlighting the need for careful resistance assessment and dedicated clinical follow-up. On the other hand, the median length of the regimens was 18 months reflecting the possibility, in the future of shorter anti-MDR and pre-XDR-TB regimens in case of localized disease, with rapid culture conversion, radiological improvement, clinical stability and good tolerance to treatment [12–20]. Regimens containing linezolid were associated with a better outcome, supporting the recent upgrade of this drug in the recent WHO guidelines [11, 19, 21]. Furthermore, meropenem/clavulanic acid containing regimens, even if burdened by intravenous administration, showed statistically significant benefits [22]. SLIs efficacy is undermined by intravenous or intramuscular administration and by high-rates of adverse events, such as nephro-toxicity, electrolyte abnormalities, pain/injury at the injection site and, importantly, vestibular toxicity and permanent ototoxicity [23–25]. Nevertheless, amikacin benefits are well known in difficult to treat TB and in fact it resists in the new WHO Group C category for DR/MDR treatment and, with different administration schedules, other then daily, side effects may be mitigated [26–28]. At the time of all-oral regimens, the use of injectable agents should be relegated in patients with no other available options on the DST [26]. Due to its difficult availability, clofazimine was introduced in antiTB regimens at Villa Marelli Institute in 2008 and therefore it was administered in a minority of patients in our study; statistically significant benefits on treatment outcome may appear with larger populations as reported in other settings [29]. FQ are very effective and relatively well tolerated against DR-TB, but resistance can rapidly develop [30]. In our study, FQ-resistance was detected in 29 (21.6%) patients and administrating FQ at standard dosage, in case of FQ-resistance at the DST, did not add any significant benefit.

Because of the high prevalence of resistance to the drugs composing the regimen and the presence of pre-XDR-TB, the WHO shorter regimen could have been administered in only 16 cases of our cohort, reaffirming the necessity of individualized regimens based on DST results in high income settings [12]. Finally, the majority of the patients (66%) received a complete outpatients diagnostic and treatment follow-up, confirming the feasibility of ambulatory care of MDR and pre-XDR-TB in appropriate settings [31, 32].

### Limitations of the study

The retrospective nature and the absence of international collaborations, in order to enlarge the study sample, are the two main limitations of the study. Even if laborious, multicentre prospective international collaboration in MDR/pre-XDR-TB treatments, in low-endemic countries, is necessary to provide more information about efficacy and tolerability of single agents composing anti-TB regimens in real life settings. The paper focus on pre-XDR-TB due to the high number of patients seen in clinical practice with these pattern of resistance, therefore XDR-TB were excluded from the analysis. Another limitation of our study is the selection of our patients according to the inclusion criteria. Our study lack the use of therapeutic drug monitoring (TDM) that is pivotal to increase efficacy and limit side effects in prolonged treatments. Unfortunately, based on the observational and retrospective nature of the study we could not assess the added value of a single drug included in the prescribed regimens. The internal validity of an observational study is poor in comparison with an experimental one; then, the findings on the effectiveness of the administered antibiotics should be proved and confirmed in larger observational or experimental studies.

## Conclusion

Tailored treatment regimens based on DST results can achieve successful outcomes in patients with pre-XDR-TB. The use of linezolid, meropenem, FQ and amikacin were linked with substantial benefit on treatment outcome in cases sensitive to those anti-TB drugs. However, MDR-TB and pre-XDR-TB remain oppressive problems, in terms of both morbidity and treatment options. Effective prevention and diagnostic strategies as well as high quality randomized trials for new MDR-TB and pre-XDR-TB regimens are needed to progress toward TB elimination.

## Data Availability

All the data are fully available upon request (mail to: luigiruffo.codecasa@ospedaleniguarda.it).

## Abbreviations

DR-TB: Drug-resistant Tuberculosis
DST: Drug-susceptibility test
EPTB: Extra-pulmonary TB
FQ: Fluoroquinolones
HIV: Human immune-deficiency virus
INH: Isoniazid
IRQ: Interquartile range
MDR-TB: Multi-drug- resistant Tuberculosis
MTB: *Mycobacterium tuberculosis*
NAAT: Nucleic acid amplification test
Pre-XDR-TB: Pre-extensively drug-resistant Tuberculosis
PTB: Pulmonary TB
RMP: Rifampicin
SD: Standard deviations
SLIs: Second-line injectable agent
TB: Tuberculosis
WHO: World Health Organization
XDR-TB: Extensively drug-resistant Tuberculosis
